# Arguments and Analogies: Do Children Have a Right to Know Their Genetic Origins?

**DOI:** 10.1002/hast.4970

**Published:** 2026-02-04

**Authors:** Sonya Charles

**Keywords:** sperm donation, anonymity, assisted‐reproductive technology, genetics, adoption, children, bioethics

## Abstract

Whether children have a right to know that they were created via “donated” gametes has generated debate for a quarter of a century. Pro‐transparency theorists use policies and attitudes concerning adoption to argue for changes in regulations related to “donor” gametes. Anti‐transparency theorists claim that discussions about whether children have a right to know their genetic origins must consider natural reproduction (and not just adoption). They argue that if we use an analogy to natural reproduction instead, we begin to see the problems with requiring transparency. I will argue that adoption is the more appropriate analogy for this debate and that we can make an argument for a strong right to know. I end with some further reasons that we can apply stronger regulation to the use of “donor” gametes than we would to natural reproduction.

## Article

Should parents tell children that they were created using “donor” gametes?^1^ This debate has been going on for over twenty‐five years. Most recently, efforts to encourage disclosure (such as open “donation” and registries for sperm “donors”) have focused on whether one has a right to know one's genetic origins. Here, I explore a specific tactic used in this debate—namely, arguing about whether the use of “donor” gametes is more analogous to adoption or natural reproduction.

Let me begin with a few clarifications. First, the question whether we have a right to know our genetic origins grows out of discussions about whether parents should tell children about the use of “donor” gametes. Conceptualizing this right is one way (but not the only way) theorists argue against deceiving “donor”‐conceived children. Second, we need to consider the relationship between a moral and a legal right. A moral right is often the basis for legal changes. Some theorists use a moral right to know one's genetic origins as a reason to outlaw anonymous gamete “donation.” To oversimplify the debate, theorists in favor of transparency believe that parents should tell children about using “donor” gametes and argue that outlawing anonymous gamete “donation” and creating registries to provide children information about “donors” is one way to encourage parents to be honest about children's genetic origins. It is true that this will not force parents to disclose the use of “donor” gametes, but these theorists believe that the ready access to information would be a nudge in the right direction. They also believe, based on a right to know, that if children find out, despite parents’ efforts to keep the information secret, that they were conceived via “donor” gametes, then the children should have access to information about the “donors.” In contrast, anti‐transparency theorists argue against these legal changes because they want to protect parents’ right to not tell children about the use of “donor” gametes.

As this very brief overview shows, the questions whether to disclose the use of “donor” gametes, whether people have a right to know their genetic origins, and whether we should enforce these moral rights by creating legal rights are—to a certain degree—intertwined. I will focus here on the uses (and abuses) of analogies related to the right to know one's genetic origins.

A significant part of the debate between these two groups turns on possible analogies. Pro‐transparency theorists use policies and attitudes toward adoption to argue for changes in regulations related to “donor” gametes. Some anti‐transparency theorists claim that discussions about whether children have a right to know their genetic origins must consider natural reproduction (and not just adoption). These anti‐transparency theorists argue that any legal enforcement of a right to know our genetic origins related to natural reproduction would be too intrusive and, if it is too intrusive in the case of natural reproduction, then it is also too intrusive in the case of assisted‐reproductive technology. Thus, understanding the strengths and weaknesses of these analogies helps in recognizing the strengths and weaknesses of the connection between a moral right to know and potential corresponding legal rights.

To make my case, I begin by explaining the stated motivations of anti‐transparency theorists who use an analogy to natural reproduction. My analysis will show why the analogy to natural reproduction is problematic and, therefore, why it does not undermine a strong right to know. I end with some further reasons that we can apply stronger regulation to the use of “donor” gametes than we would to natural reproduction. Ultimately, I claim that using “donor” gametes is more analogous to adoption than natural reproduction because it requires third‐party assistance. As third parties should not be complicit in perpetuating a deception, anonymous gamete “donations” should not be allowed.

## What Is at Stake?

There is some variation in how much those who support transparency argue explicitly for a right to know one's genetic origins versus focusing on potential emotional harm or some other reason for transparency. However, since anti‐transparency theorists focus on this presumed “right to know,” I will generally use this language as well. Pro‐transparency theorists also argue for a variety of practical proposals, but the most relevant to the current discussion is the creation of some sort of database that would record the use of “donor” gametes and allow “donor”‐conceived children access to identifying information when they reach adulthood.^2^ In effect, these proposals argue for outlawing anonymous gamete “donation” and allowing children access to information about their origins. Anti‐transparency theorists see this as an unwarranted intrusion into the parent‐child relationship. To focus the following discussion, I will draw on the arguments made by Glenn Cohen and by An Ravelingien and Guido Pennings. All these authors consider how we could apply the right to know one's genetic origins to natural reproduction and use this comparison to undermine a strong right to know. Before getting into the specific analogies, let us take a brief look at their motivation.

In “Rethinking Sperm Donor Anonymity,” Cohen argues that, when thinking about families created using “donor” gametes, we should consider not only the analogy to adoption but also the analogy to natural reproduction.^3^ He maintains that if we apply a right to know to those who use “donor” gametes but not natural reproduction, then we are participating in “reproductive‐technology exceptionalism.”^4^ It is unfair, in his view, to apply a right to know only to “donor”‐conceived children and not to also apply it to those who are the result of coital reproduction.

In “The Right to Know Your Genetic Origins,” Ravelingien and Pennings state the issue even more forcefully, insisting that “*if* ‘donor’‐conceived children have a fundamental right to know their genetic origins, it would be unjust and discriminatory not to extend this right to all children with uncertain or unknown genetic backgrounds. This means that all children who were conceived naturally should be informed about the genetic relationship with their social father, because paternity is always uncertain.”^5^ Like Cohen, they go on to consider what it would take to enforce this right in the case of natural reproduction. Also like Cohen, they believe that an analogy to natural reproduction undermines the right to know (and, by implication, proposals that eliminate anonymity for gamete “donors”).

Cohen and Ravelingien and Pennings alike propose ways to enforce a right to know in relation to natural reproduction. To be clear, these are not serious proposals. The authors recognize that most will find these proposals outrageously intrusive. Their point is that, if we find these too intrusive, then we should find proposals to outlaw anonymous gamete “donation” equally intrusive. Recall that Cohen refers to the disparate arguments as “reproductive‐technology exceptionalism.” However, I believe that Cohen's and Ravelingien and Pennings's arguments are ineffective because they ignore the biggest disanalogy between assisted‐reproductive technology (ART) and natural reproduction—the inclusion of third parties in the process. This is my main reason for arguing that adoption is a better analogy than natural reproduction for the use of gamete “donation.” Before getting into this argument, I consider each proposal in turn to show why the analogies drawn by Cohen and by Ravelingien and Pennings fail. Since the strength of their criticism rests on the analogy, criticism of their analogy also undermines their critiques. By showing the weaknesses in their objections to transparency, I hope to strengthen the policy recommendations of outlawing anonymous gamete “donation” and allowing “donor”‐conceived children access to identifying information.

## The Proposals: Sex Registries and Paternity Testing

Responding to the argument that we should have a national sperm registry so that “donor”‐conceived children can access information about their “donors,” Cohen makes what he calls a “Swiftian” proposal: “[W]hy not require every individual who engages in coital sex with a fixed probability of conception to put his or her name and contact information in a registry mirroring the [sperm donor registry] endorsed by [Naomi] Cahn, with the proviso that their names would be erased from the registry if there were no evidence of pregnancy within a few months? This would encompass a ‘one‐night‐stand registry’ in that never again would a child who results from a temporary relationship suffer the harms which Cahn envisions and which she claims call out for governmental regulation.”^6^ Cohen admits his proposal is meant to provoke. In putting forth a proposal that most would find abhorrent, he hopes to show that children do not really have a right to know their genetic origins. If we are not willing to secure this right for children who are naturally conceived, then how do we justify interfering with parents who use ART?

Cohen's proposal is, of course, far more intrusive than a national registry of gamete “donors” would be. His proposal would apply to all heterosexual couples with the potential to reproduce, regardless of whether intercourse led to pregnancy. The obligatory registry would constitute an unwarranted intrusion into intimate sexual relationships, whereas proposals related to gamete “donations” target the parent‐child relationship. In general, the state has more leeway in regulating the parent‐child relationship than it does to police intimate relations between consenting adults. So, the first major problem with this analogy is that the intervention intrudes on the wrong relationship.

At first glance, Ravelingien and Pennings's proposal appears to be directed at the correct relationship (parent‐child); however, it is in fact also overly intrusive. Instead of a sex registry, Ravelingien and Pennings argue in favor of mandatory paternity testing at birth: “[W]e wish to put forward a realistic and feasible scenario that could theoretically ensure universal respect for this right: one in which DNA paternity testing is routinely performed on the newborns of heterosexual couples…. [T]his would be a practical means to ensure that each instance of false paternity is intercepted and that all children are given an equal chance to learn the truth about their genetic origin.”^7^ Their intentionally provocative proposal aims to show the weakness in a right‐to‐know argument. Paternity testing to ensure accurate recording of genetic origins would be one way to secure a child's right to know. And, if it affected only the parent‐child relationship, it would probably be justified. Yet this proposal also has implications for the intimate relationship between adults.

When the test confirms paternity of the male partner in a case of natural reproduction, there is no problem. However, if the paternity test shows that the male partner is not the father (presumably the reason for having mandatory testing), then the moral wrong uncovered will relate to the intimate relationship between adults. Further, in the case of natural conception, the woman is likely already participating in unethical behavior. Cheating on one's spouse or partner is not usually seen as a moral good. Lying about this behavior on the child's birth registry arguably compounds the moral wrong. In contrast, there is nothing inherently immoral in using “donor” gametes—in this case, the lie is the main moral wrong. Pinpointing the moral wrong highlights a key difference in these cases. In the case of natural reproduction, there are two moral wrongs—first, infidelity, which is a moral wrong against the intimate partner, and, second, a lie about paternity, which is a moral wrong against the child (and the partner). In the case of ART, the parents collude in the same moral wrong against the child by lying about the child's genetic origins.^8^

Again, we can recognize that children have a right to know their genetic origins and that any woman who lies about their child's father in cases of natural reproduction has committed a moral wrong. But the reason mandatory paternity testing is too intrusive is because of its effects on the adult relationship (not the parent‐child relationship). Indeed, as many point out, there can be other real harms to women by implementing this policy.

In “Whose Right to Know?,” Erin Heidt‐Forsyth and Michelle McGowan respond to Ravelingien and Pennings's argument for mandatory paternity testing primarily by noting that it ignores the woman and her social position. They remind us that, in the United States, a precedent for mandatory paternity identification can be found in the way the state implements the social safety net. The 1996 Personal Responsibility and Work Opportunity Reconciliation Act included mandatory reporting of paternity for children receiving aid. “To enforce the social role of fathers, the federal government—with participation of states—requires recipients of TANF [Temporary Assistance for Needy Families] to identify their child's father, and to assist in collecting child support from him.”^9^ As Heidt‐Forsyth and McGowan point out, this policy is not only intrusive but also opens women (and children) to potential harm. “Gwendolyn Mink and others argue that mandatory paternity identification threatens the safety of woman and children, compels mothers to create association between fathers and children, and exposes women to private violence that can result from paternity identification,” Heidt‐Forsyth and McGowan explain.^10^ Surely, making children (and their mothers) vulnerable to potential intimate violence is a significant‐enough harm to rule out mandatory paternity testing or identification.

While I strongly disagree with tying state aid to paternity requirements, this policy example is useful in explaining my criticisms of Cohen's and Ravelingien and Pennings's proposals. First, the state has more leeway to regulate the parent‐child relationship than it does to interfere with intimate relationships between adults. Even though we may not like the policy itself, the state justifies mandatory paternity identification as a way to get men to live up to their parental responsibilities. Second, the government has more justification for regulation when third parties are involved in the parent‐child relationship. The government does not track down all single mothers and demand paternity identification. They implement this policy when the state is asked to fill in for the missing father in the form of economic support. To be clear, I am not advocating for state policies that reinforce traditional gender roles. I use this example only to show why adoption is the more appropriate analogy for ART. Unlike natural reproduction, both adoption and the use of “donor” gametes bring third parties into the process of family formation. Moreover, policies promoting open adoption and national registries for gamete “donors” focus on the parent‐child relationship. If they are intrusive, the intrusion is into the parent‐child relationship (not the intimate relationships between adults).

## Justifying Intervention, Regulating Markets

From the arguments discussed above, we can see that anti‐transparency theorists equivocate between a moral and a legal right. We could summarize Ravelingien and Pennings's argument with these three sentences: If there is a (moral) right to know one's genetic parents, then this right to know must apply equally to all. However, if we consider how we would (legally) apply this to naturally conceived children, then we see that the laws would be unduly intrusive into the parent‐child relationship. Therefore, there is no (moral) right to know one's genetic parents. Ravelingien and Pennings thus use the intrusiveness of the legal remedy to argue against the moral right.

Whenever you are making a moral argument, there are two different considerations: Is X behavior or action moral or immoral? And what can or should we do to encourage or force people to behave morally? There is nothing inconsistent in saying that X is morally wrong but that we cannot really do anything to enforce not‐X because that would lead to other wrongs. A classic example I often use with my students is cheating on your significant other. If you are in a sexual relationship and the understanding between you and your partner is that this is a monogamous relationship, then it is immoral to have sex with someone outside of that relationship. While most would agree with this analysis, most would also agree that we do not wish to make laws against cheating. We do not believe that it is appropriate to bring state power into the relationship in this way because it would likely lead to other wrongs, such as state violation of the right to privacy. In sum, we need to separate arguments about what is moral and immoral from those about what we can do to enforce morality. We can argue that children have a right to know their genetic origins but have a separate argument about what policies we can or cannot use to support this right.

As I said before, when you bring third parties into the process of family creation, then you open yourself up to greater oversight. Anti‐transparency theorists say that, when considering “donor”‐conceived children, natural coitus is just as valid an analogy as adoption, and they argue that legal rights to know genetic parents in relation to naturally conceived children are too intrusive. They then use the intrusiveness of legal remedies related to natural reproduction to undermine *any* moral right to know one's genetic parents. Yet none of these theorists argue against legal changes that allow adopted children access to information about their biological parents. Why not? If there is no moral right to know your genetic parents, then should we not also get rid of laws that allow adopted children access to information about their genetic parents? I will return to this discrepancy later. For now, I argue that these theorists’ analogies show only that it is more difficult to enforce this moral right when reproduction occurs naturally than it is when third parties are involved in the procreative process.

So far, I have argued that adoption is a better analogy than natural reproduction because the use of third parties in procreation opens one up to more regulation, but we can take this argument one step further. When couples use “donor” gametes, they are not only bringing third parties into the process of procreation; they are also participating in a market—and we generally agree that it is acceptable for governments to regulate markets. Of course, no one likes to admit that infertility services are a market, but they clearly are markets. As Deborah Spar puts it in *The Baby Business: How Money, Science, and Politics Drive the Commerce of Conception*, “[W]e have markets in all kinds of things, including health care, childcare, and education, and we generally think they work. We have a demand for babies, a supply of babies, and a growing set of intermediaries who serve to bring demand and supply together. Given this situation, we essentially have two choices. We can rue the baby business and call for its demise, or we can accept the market that has arisen and try to make it better.”^11^ She makes a strong argument in favor of regulation to bring order to the system. Specifically, we need to clarify who has rights to what and the circumstances under which these rights apply. This is precisely what those who propose more transparency in the use of “donor” gametes are trying to do.

Despite the euphemism of “donor,” most gametes are procured through buying and selling—which are market activities and thus open to regulation. Returning to the discussion of what specific policies and regulations are justifiable, I doubt anyone would favor doing away with regulations that require sperm banks to test for HIV and other diseases before offering sperm for sale. Yet we would find it outrageous to require all couples to undergo disease testing before having sex. This reinforces both the idea that it is okay for states to regulate markets and that some intrusions are justified, but others are not. If a business is going to pay one person to obtain gametes and charge another to use those gametes, then the state has the authority to put regulations on how these transactions proceed. Again, the justification is twofold. Pro‐transparency policies are directed at the parent‐child relationship (not intimate relationships between consenting adults), and use of “donor” gametes is part of an ART market—which is open to government regulation.

Let me also say a few words about Cohen, Ravelingien, and Pennings's concern that, if this is a right, it should be uniformly enforced. Again, there is a difference in arguing about what is moral or immoral and about what we can or should do to enforce moral actions. This distinction applies both to natural reproduction and to the use of “donor” gametes. Given that some people participate in sperm “donation” via more informal transactions, it leaves open the question of how far the state should or could go in regulating these situations. My arguments apply mainly to commercial transactions via fertility clinics. My initial thought is that noncommercial situations would not be subject to the same regulations (and in that way would be more like natural reproduction), but these informal situations would also not have the same regulatory protections that are written into public policies (such as requests for noncontact and protection from parental obligations). However, I leave open the possibility of whether we would want to make other laws related to these informal transactions in the same way we outlaw baby selling, selling organs, and so forth.

Again, the parallels to adoption seem clear. There are certainly situations in which children are raised mainly by someone other than their biological parents (such as grandparents or siblings) even though that relationship is never formalized via an official adoption process. These situations leave children and their caregivers vulnerable, even though there may be a variety of reasons that they are never formalized. In contrast, in formal adoption procedures, the government has specific policies and regulations in place (presumably) to protect children, biological parents, and adopting parents. The government has an interest in protecting children and making sure people are not participating in behaviors that we find reprehensible, such as baby selling or human trafficking. Note that those using ART are already privileged in that there is usually minimal to no vetting of those using the service (unlike with adoption, which requires prospective parents to go through a vetting process). In sum, the use of commercial transactions and third‐party involvement in procreation means that ART is more like adoption than like natural procreation; therefore, the arguments put forth by critics of transparency about gamete “donation” are unpersuasive.

## Further Objections

While offering a comprehensive argument in favor of outlawing anonymous sperm “donation” is beyond the scope of this paper, here, I address some further objections to my pro‐transparency argument.

As I mentioned at the start, anti‐transparency theorists believe that the policies supported by pro‐transparency theorists are too restrictive of parental choice. For example, Cohen rejects a “one‐size‐fits all” solution because it interferes with people's ability to “maximize their own welfare and life plans.”^12^ Ravelingien and Pennings likewise reject a “one‐size‐fits‐all” solution and policies in which “the government intrudes into one's intimate and private life.”^13^ Similarly, in *Conceiving People: Genetic Knowledge and the Ethics of Sperm and Egg Donation*, David Groll considers the question of parental failures. He believes that deceiving children about their genetic origins is a significant parental failure, but not one that requires legal remedy. He argues that it is no worse than other parental failures that we do not find worthy of legal intervention. Even though he does not use the same language as Cohen, Groll ultimately argues that laws forcing parents to disclose the use of “donor” gametes would be a kind of reproductive exceptionalism. “The law,” he states, “says to *this* subset of intended parents what it does not say to other parents, namely: ‘This particular kind of parental failure must be prevented.’ Traditional parents are given wider discretion to parentally fail than non‐traditional parents.”^14^

I disagree about the level of “parental failure” involved in deceiving children about their genetic origins. Elsewhere I have argued that lying to children about their genetic origins is a breach in trust between parents and children. I refer to this as a kind of “foundational lie” that undermines the relationship.^15^ In addition, research shows that children who find out later about being “donor”‐conceived have greater psychological issues and often feel betrayed by their parents.^16^ Throughout this paper, I have argued in favor of a moral right to know one's genetic origins while simultaneously considering which corresponding legal rights are justified. Given that Groll's main objection is to the legal remedies, let us take a closer look at his argument.

Even though he is mostly in favor of transparency, Groll also argues that outlawing anonymous sperm “donors” is too intrusive because it would require forcing parents to disclose the nature of the child's conception. Groll admits that outlawing the use of anonymous “donors” seems like an easy, unobtrusive fix. However, Groll (and others) point out that this does not fully address a child's right to know their genetic origins. Heterosexual parents could still lie about or conceal the nature of the child's conception, and, thus, the child would never know to request information about their genetic parent(s). Yet Groll argues that requiring parents to disclose this information makes the legal remedy too intrusive. So, like other theorists, Groll seems to be implying that, as outlawing anonymous “donation” does not fully remedy the situation, it is not worth doing at all.

My response here is twofold. First, governments and businesses should not be complicit in helping parents perpetuate this deception, and outlawing anonymous “donation” addresses this component. Second, analyzing ART as a market, Spar argues that we can justify some limits on parental choice because (cumulatively) these decisions will shape society. So, if we agree that children have a moral right to know their genetic origins, then a national registry of gamete “donors” might be a first step in recognizing that as a legal right—even if we are not ready to take further steps to guarantee this right. Society would move toward recognizing and supporting this moral right in an incremental way.

Finally, we should consider whose interests are protected and harmed in keeping or outlawing anonymous sperm “donors.” Why is it that none of the theorists who oppose outlawing anonymous sperm “donors” argue in favor of undoing laws that allow adopted children access to information about their genetic parents? Very likely, in many cases, it is because the person who maintains that these laws are too intrusive is mostly interested in protecting heterosexual privilege. As many have pointed out, a right to know your genetic origins is a twofold right—a right to know certain circumstances of your conception and a right to information about your genetic parent(s). Only heterosexual parents can lie or deceive a child about certain circumstances of their conception. In single‐parent and same‐sex‐parent families, it will be obvious that part of a child's genetic heritage comes from outside the family unit. For this reason, I believe that those who most vehemently argue against outlawing anonymous sperm “donors” are mainly interested in protecting heterosexual privilege.

Same‐sex couples sometimes prefer anonymous donors because they fear genetic parents will challenge their parental rights. However, even when same‐sex couples use anonymous gametes, many often feel guilty that the children will not be able to know the identity of their genetic parents later in life.^17^ Same‐sex families are put in a bind. The nature of their situation means that their children will know part of their genetic heritage came from somewhere else. These parents do not have the privilege of keeping this information secret. Yet the right of same‐sex couples to be parents is a precarious right—especially when there is no genetic connection. If a woman married to a man gives birth using “donor” sperm, all states will automatically recognize her husband as the legal coparent. By contrast, if a woman married to another woman gives birth using “donated” sperm, her wife may still have to adopt the child to be recognized as a legal parent.^18^ When using a known donor, the couple is thus left vulnerable to challenges from the “donor,” should he want to assert parental rights. For these reasons, same‐sex couples sometimes prefer anonymous “donors” but regret that the children will not have access to information about the “donor” later in life.

Getting rid of anonymous “donors” could potentially burden same‐sex couples in other ways as well. Given that they cannot deceive their children into believing that they are the biological offspring of both parents, their children will know about the existence of a “donor.” Although some research shows that children in lesbian couples are less interested in specific information about their genetic parents than are kids in heterosexual families,^19^ children of same‐sex couples could seek out the “donor.” These couples may consequently face intrusions from a “donor” that heterosexual couples can avoid. This problem could be addressed by writing the laws to restrict access until children reach the legal age of majority.

A more practical problem is that outlawing anonymous “donors” often leads to a decrease in the availability of “donors.” This problem would limit options for same‐sex couples and would especially impact people of color, disproportionately affecting already marginalized communities. Fully addressing this problem is beyond the scope of this paper, but I will provide some initial thoughts. The problem immediately raises questions about whether prospective parents have the right to an adequate supply of third‐party gametes. We would need to weigh a right or desire for third‐party gametes against a child's right to know or interest in knowing their genetic origins. As Groll points out, parents turn to ART because they value having at least one parent be genetically connected to the child—which implies that genetic ties are important enough for us to realize that the “donor”‐conceived child might have a significant interest in knowing about their genetic origins.^20^ The interests of “donor”‐conceived children in having access to that information are equal to the interests parents have in at least one parent being genetically tied to their child. One way to mediate the decrease in market supply would be to create better legal protections for those who want to use known donors—especially since protecting their legal rights is one reason that same‐sex couples seek out anonymous “donors.”

The final objection I would like to briefly address is whether arguments related to a right to know one's genetic origins reinforce the idea of bionormativity—the idea, that is, that only genetically related parents are “real” parents. Some, like Groll, argue that we need to first work to undermine bionormativity—otherwise, outlawing anonymous gamete “donation” might reinforce it.^21^ I disagree. First, if we return to our analogies, we see that theorists who argue against outlawing anonymous gamete “donation” (including Groll) do not also argue in favor of resealing adoption records. In the case of adoption, it seems to be easier to recognize that children's curiosity about their biological origins does not mean that they are rejecting their social parents or do not see their social parents as their “real” parents. Second, as we have seen, many theorists who argue against anonymous gamete “donation” are really interested in protecting heterosexual privilege or heterosexual parents’ ability to conceal the use of “donor” gametes. To his credit, Groll is not making this kind of argument. My disagreement with Groll is more of a chicken‐or‐egg problem. He worries that outlawing anonymous gamete “donation” before undoing bionormativity will reinforce bionormativity. In contrast, I would argue that polices and regulations that normalize various approaches to family formation work to broaden our definition of family. If we acknowledge that using “donor” gametes is just one, among many, ways to procreate, then this broadens our definition of a “normal” family. Codifying access to information about biological parents (for both adopted and “donor”‐conceived children) not only protects the interests of children but also normalizes a variety of ways to create families by officially acknowledging their existence. Again, this is a *first* step in creating new norms.
